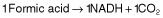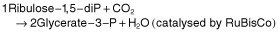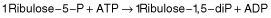# Kinetic and stoichiometric characterization of organoautotrophic growth of Ralstonia eutropha on formic acid in fed-batch and continuous cultures

**DOI:** 10.1111/1751-7915.12149

**Published:** 2014-08-13

**Authors:** Stephan Grunwald, Alexis Mottet, Estelle Grousseau, Jens K Plassmeier, Milan K Popović, Jean-Louis Uribelarrea, Nathalie Gorret, Stéphane E Guillouet, Anthony Sinskey

**Affiliations:** 1 Department of Biology, Massachusetts Institute of TechnologyBldg. 68-370, 77 Massachusetts Avenue, Cambridge, MA, 02139, USA; 2 Division of Health Sciences and Technology, Massachusetts Institute of TechnologyBldg. 68-370, 77 Massachusetts Avenue, Cambridge, MA, 02139, USA; 3 Engineering Systems Division, Massachusetts Institute of TechnologyBldg. 68-370, 77 Massachusetts Avenue, Cambridge, MA, 02139, USA; 4 Department of Biotechnology, Beuth Hochschule für Technik Berlin13353, Berlin, Germany; 5 Université de Toulouse; INSA, UPS, INP; LISBPF-31077, Toulouse, France; 6 INRA, UMR792 Ingénierie des Systèmes Biologiques et des ProcédésF-31400, Toulouse, France; 7 CNRS, UMR5504F-31400, Toulouse, France

## Abstract

Formic acid, acting as both carbon and energy source, is a safe alternative to a carbon dioxide, hydrogen and dioxygen mix for studying the conversion of carbon through the Calvin–Benson–Bassham (CBB) cycle into value-added chemical compounds by non-photosynthetic microorganisms. In this work, organoautotrophic growth of *Ralstonia eutropha* on formic acid was studied using an approach combining stoichiometric modeling and controlled cultures in bioreactors. A strain deleted of its polyhydroxyalkanoate production pathway was used in order to carry out a physiological characterization. The maximal growth yield was determined at 0.16 Cmole Cmole^−1^ in a formate-limited continuous culture. The measured yield corresponded to 76% to 85% of the theoretical yield (later confirmed in pH-controlled fed-batch cultures). The stoichiometric study highlighted the imbalance between carbon and energy provided by formic acid and explained the low growth yields measured. Fed-batch cultures were also used to determine the maximum specific growth rate (μ_max_ = 0.18 h^−1^) and to study the impact of increasing formic acid concentrations on growth yields. High formic acid sensitivity was found in *R* *eutropha* since a linear decrease in the biomass yield with increasing residual formic acid concentrations was observed between 0 and 1.5 g l^−1^.

## Introduction

*Ralstonia eutropha*, also known as *Cupriavidus necator*, is an aerobic facultative autotrophic bacterium, able to convert carbon dioxide (CO_2_) or formic acid (HCO_2_^−^) through the Calvin–Benson–Bassham (CBB) cycle into value-added chemical compounds, such as polyhydroxyalkanoates (PHAs) (Ishizaki and Tanaka, 1991) or biofuels (isobutanol, 3-methyl-butanol, isopropanol) (Li *et al*., 2012; Lu *et al*., 2012; Grousseau *et al*., 2014). When CO_2_ is used as unique carbon source, an energy source must be supplied: *Ralstonia eutropha* is able to utilize inorganic hydrogen (H_2_) in combination with dioxygen (O_2_) as electron donor and acceptor respectively. However, the use of H_2_ and O_2_ requires an exact control of the gas composition. Although strategies to prevent mixed-gas explosion have been successfully developed, even for high cell density fermentations (Tanaka *et al*., 1995), they require additional expensive safety measures concerning staff and facilities.

As a safe alternative to H_2_, formic acid can be used as energy and carbon source during organoautotrophic growth (Li *et al*., 2012) and can be electrochemically produced from CO_2_ and H_2_O (Udupa *et al*., 1971; Ikeda *et al*., 1987) offering the opportunity to valorize waste CO_2_. Oxidation of formic acid catalysed by a formate dehydrogenase delivers one nicotinamide adenine dinucleotide reduced (NADH) and one CO_2_, which can be assimilated via the CBB cycle (Bowien and Schlegel, 1981), as it is the case during lithoautotrophic growth.

In this context, formic acid is a substrate of high interest. Studies aiming to improve lithoautotrophic growth of *R. eutropha* were reported: (i) high-cell densities up to 25 g cell dry weight (CDW) l^−1^ have been reached, by carefully investigating the macro- and micronutrient requirements of *R. eutropha* (Repaske and Repaske, 1976); (ii) up to 91.3 gCDW l^−1^ have been reached by developing special agitation systems and adjusting the gas composition (Tanaka *et al*., 1995). However, few studies describe organoautotrophic growth of *R. eutropha* and those focus either on formic acid metabolism (Friedrich *et al*., 1979) or proteomic examination of *Ralstonia* in response to formic acid (Lee *et al*., 2006). Stoichiometric and kinetic characterization of *R. eutropha* growth on formic acid as the sole carbon and energy source was generally neglected, and only very low biomass concentrations (about 1.2 to 1.7 gCDW l^−1^) have been reached (Friedrich *et al*., 1979; Friedebold and Bowien, 1993; Li *et al*., 2012).

It is well known that short-chain organic acids including formic acid are toxic to cells (Salmond *et al*., 1984; Pronk *et al*., 1991; Russell, 1992; Vazquez *et al*., 2011). However, toxicity of formic acid to *R. eutropha* was rarely explored. It has been shown in pulse-fed flask cultures, initially grown on 2 g l^−1^ of glucose, that the biomass yield of *R. eutropha* decreases with increasing concentrations of formic acid (Lee *et al*., 2006).

Since formic acid is toxic, a batch culture with this substrate is not a suitable culture system. Usually, a pH-controlled feeding (pH-stat) strategy is used for organoautotrophic growth of *R. eutropha* on formic acid (Friedebold and Bowien, 1993; Li *et al*., 2012).

This study aimed at determining the maximum growth capacities of *R. eutropha* (i.e. rate and yield) on formic acid as a sole substrate and at investigating the impact of increasing formic acid concentrations. Therefore, two different culture systems were applied to characterize the growth of *R. eutropha* on formic acid as a sole substrate:

- A chemostat culture was performed to determine the maximal yield with no residual formic acid concentration.
- pH-controlled fed-batch cultures, designed to maintain concentrations of formic acid between 0 and 2 g l^−1^, were performed to confirm the maximal biomass yield determined with the chemostat system and to investigate the effect of increasing concentrations of formic acid on the biomass yield. The fed-batch culture as a dynamic system was also used to study the growth kinetics.

Moreover, experimental results were compared to theoretical results from stoichiometric modeling.

## Results and discussion

### Biomass concentrations produced from formic acid

The organoautotrophic growth of a *R. eutropha*-engineered strain deficient in polyhydroxybutyrate (PHB) production (Re2061; Lu *et al*., 2012) was investigated in pH-controlled fed-batch and continuous cultures using well-designed medium and fully equipped bioreactors. In those conditions, the final biomass concentration reached 5.4 gCDW l^−1^ in the pH-controlled fed-batch cultivation and 10.6 gCDW l^−1^ in the chemostat with formic acid as the sole carbon and energy sources. These biomass concentrations are the highest ever published (Table 1). The first detailed study for growth of *R. eutropha* on formic acid in a pH-controlled fed-batch fermentation (10 l) was performed by Friedrich and colleagues (1979) with the wild-type strain H16 able to produce PHB. A maximal biomass concentration of 1.2 gCDW l^−1^ was reached (Table 1). In a publication that focused on the characterization of the soluble formate dehydrogenase of *R. eutropha*, approximately 1.7 g l^−1^ of CDW was reached (Table 1) during a 10 l fed-batch fermentation (Friedebold and Bowien, 1993). In these two articles, the same basal media was used (Schlegel *et al*., 1961), and addition of a second trace solution (SL7) was performed by Friedebold and Bowien (1993). Some nutrient limitations may have occurred: the nitrogen amount corresponded to the amount necessary to produce about 1.9 gCDW l^−1^ of biomass considering the following biomass formula: C_1_H_1.77_O_0.44_N_0.25_ (4% ashes) and a molecular weight of 25.35 g Cmole^−1^ (Aragao, 1996). Moreover, in Friedrich and colleagues (1979), an O_2_ starvation was thought to be the reason for the cessation of cell growth at a CDW of 1.2 g l^−1^ since a higher biomass concentration was reached under lithoautotrophic condition. In a recent publication that focused on the electrochemical production of formic acid, a fed-batch fermentation was performed as a side experiment in a 5 l reactor with a strain unable to produce PHB (Li *et al*., 2012). A biomass concentration of 1.4 gCDW l^−1^ (Table 1) was reached in accordance with the media composition: the nitrogen concentration of 0.015 mole l^−1^ corresponded to the amount necessary to produce about 1.5 g l^−1^ of CDW.

**Table 1:** Organoautrophic biomass production with *R. eutropha*

Strain	Phenotype	CDW_max_ (g l^−1^)	Culture type	Reference
*R. eutropha* Re2061	PHB^−^	10.5	chemostat	This work
*R. eutropha* Re2061	PHB^−^	5.4	fed-batch	This work
*R. eutropha* H16	wild type	1.2	fed-batch	Friedrich *et al*., 1979
*R. eutropha* H17	wild type	1.7^a^	fed-batch	Friedebold and Bowien 1993
*R. eutropha* LH74D	PHB^−^ isobutanol^+^	1.4^b^	fed-batch	Li *et al*., 2012

a The CDW was not estimated in Friedebold and Bowien (1993), an OD_436_ of 8 was measured which corresponded to a CDW of approximately 1.7 g l^−1^ (1 g CDW l^−1^ corresponding to an OD_436_ of 4.8).

b The CDW was not estimated in Li *et al*. work, an OD_600_ about 3.8 was measured. Using the ratio CDW/OD_600_ = 0.363 g l^−1^. UDO^−1^ determined in this work [calibration curve done with 24 data points from fed-batch cultures, OD measured using a 1 cm path length absorption PS semi-micro cuvette (VWR, Radnor, PA, USA) with a Spectronic GENESYS 20 Visible Spectrophotometer at a wavelength of 600 nm] and assuming that the authors were using a 1 cm path length cell for measurement, the equivalent CDW was about 1.4 g l^−1^.

The high biomass concentrations reached in this work enabled to perform a reliable quantification of the *R. eutropha* growth kinetics and stoichiometry on formic acid.

### Determination of the maximal biomass production yield in formic acid limited continuous culture

A continuous culture of *R. eutropha* was performed at a low dilution rate of 0.05 h^−1^ and under formic acid limitation.

The continuous cultivation data are presented in Table 2. The results were obtained by averaging data over a period of 20 h after reaching the steady state. The steady state was considered to be reached when the standard deviation of the variation of biomass concentration, CO_2_ production rate and O_2_ consumption rate was inferior to 1%. The biomass concentration was maintained at a value equal to 10.58 ± 0.07 gCDW l^−1^. No residual formic acid concentration was detected during the steady state, and no other metabolites were detected. A biomass yield on formate of 0.16 ± 0.00 Cmole Cmole^−1^ was achieved. Carbon and reduction degree balances were respectively equal to 98.2 ± 0.4% and 103.1 ± 0.1% (Table 2) confirming that no other products than biomass were produced from formic acid.

**Table 2:** Experimental and theoretical data concerning chemostat culture of *R. eutropha*

	CDW	qO_2_	qCO_2_	qH_2_		C balance	Redox balance	D = μ	Ys/x	qS
	g l^−1^	mmol g^−1^ h^−1^	mmol g^−1^ h^−1^	mmol g^−1^ h^−1^	RQ	%	%	h^−1^	Cmole Cmole^−1^	Cmole g^−1^ h^−1^
Formic acid as substrate										
Experimental data	10.59 ± 0.07	4.01 ± 0.03	10.09 ± 0.05	×	2.52 ± 0.03	98.2 ± 0.4	103.1 ± 0.1	0.05 ± 0.00	0.16 ± 0.00	12.05 ± 0.05
Theoretical data	×	3.92	10.16	×	2.59	100	100	0.05^a^	0.16^a^	12.17
CO_2_ as substrate										
Theoretical data	×	3.14	− 2.02	10.61	− 0.65	100	100	0.05^a^	0.97	− 2.02

a Value set in the stoichiometric model.

**Figure 1:**
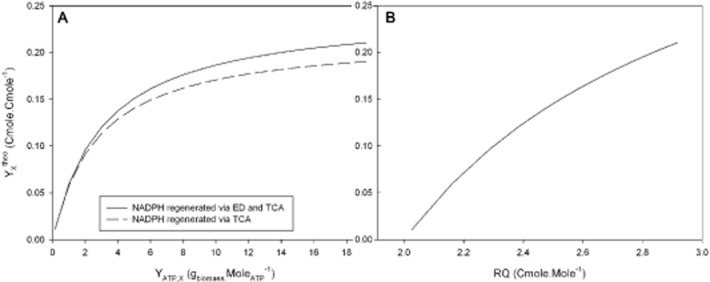
Stoichiometric modeling results.A. Simulation of theoretical biomass production yield (Y_X_^theo^) on formic acid depending on energetic yield (Y_ATP,X_) with two NADPH production pathway (Entner Doudoroff (ED) and Tricarboxylic acid cycle (TCA)).B. Simulation of theoretical biomass production yield (Y_X_^theo^) on formic acid depending on respiratory quotient (RQ).

The stoichiometric model constructed by Grousseau and colleagues (2013) was implemented as explained in *Calculations and metabolic descriptor* in order to compare theoretical and experimental data. Theoretical biomass production yields were calculated depending on the energetic yield (Y_ATP,X_) and NADPH production pathway [Entner–Doudoroff (ED) or tricarboxylic acid cycle (TCA)] as depicted on Fig. 1A.

The maximum Y_ATP,X_ of 19.21 g_biomass_ mol_ATP_^−1^ is the anabolic demand in adenosine triphosphate (ATP) calculated according to the biomass composition and the anabolic reactions. The corresponding biomass theoretical yield is between 0.19 and 0.21 Cmole Cmole^−1^ depending on the NADPH generation pathway (Fig. 1A). The experimental biomass yield of 0.16 ± 0.00 Cmole Cmole^−1^ obtained in chemostat condition corresponds to 76% to 84% of this maximal theoretical yield. The maximal theoretical value of Y_ATP,X_, calculated from the energetics of the anabolic pathways, is generally more than twice the experimental yield (Neijssel and Demattos, 1994). This difference can be explained by futile cycle, protein and nucleic acid turnovers and by useful maintenance (ionic transports, cellular homeostasis). These extra ATP requirements were included in the model by an ATP spilling reaction (Grousseau *et al*., 2013). According to Fig. 1A, the experimental biomass yield of 0.16 Cmole Cmole^−1^ corresponds to a Y_ATP,X_ between 6 and 8 g_biomass_ mol_ATP_^−1^, in accordance with Neijssel and Demattos (1994) assumption.

The experimental biomass yield was associated to an experimental respiratory quotient (RQ) of 2.52 ± 0.03 which is very closed to the theoretical value of 2.59 (Table 2 and Fig. 1B).

During the continuous culture, specific dioxide carbon production rate (qCO_2_) and dioxygen uptake rate (qO_2_) were calculated (Table 2). The theoretical qCO_2_ and qO_2_ were also numerically simulated using the stoichiometric model and were equal to 10.16 and 3.92 mmol g^−1^ h^−1^ respectively. These values were very closed to the experimental data with 10.09 ± 0.05 mmol g^−1^ h^−1^ for qCO_2_ and 4.01 ± 0.03 mmol g^−1^ h^−1^ for qO_2_ (Table 2).

The biomass yields obtained for growth on formic acid appeared to be low, compared to other substrates like fructose with 0.63 Cmole Cmole^−1^ (fed-batch culture with strain Re2061, data not shown), or butyric acid with 0.65 Cmole Cmole^−1^ (Grousseau *et al*., 2013), or pyruvate with 0.53 Cmole Cmole^−1^ (Friedrich *et al*., 1979), or CO_2_ with 0.92 Cmole Cmole^−1^ (Morinaga *et al*., 1978). This could be explained by the low efficiency of the formic acid to provide NADH (only 1 mole of NADH per mole of formic acid) compared to the high requirement of the CBB cycle to assimilate CO_2_ derived from the oxidation of formic acid: 1.7 NADH and 2.7 ATP per CO_2_ (3 CO_2_ + 5 NADH + 8 ATP + 5 H_2_O → 3-P-Glycerate + 8 ADP + 5 NAD^+^). Considering the respiratory chain used by Grousseau and colleagues (2013), 1.35 NADH are necessary to produce 2.7 ATP. The requirement of the CBB cycle is therefore equivalent to three NADH per CO_2_. Since the oxidation of formic acid delivers one NADH and one CO_2_, three moles of formic acid are required for the fixation of one mole of CO_2_ by the CBB cycle, leading to a maximal yield of only 0.33 Cmole Cmole^−1^. The consideration of the whole reaction system to produce biomass in organoautotrophic condition leads to a value between 0.19 and 0.21 Cmole Cmole^−1^ given above. When the energy source (H_2_) is dissociated from the carbon source (CO_2_) in lithoautotrophic condition, the biomass production yield is close to 1 Cmole Cmole^−1^ according to the modelling (Table 2) and in accordance with Morinaga and colleagues (1978). The high NADH requirement of the CBB cycle affects, in this case, the biomass yield on H_2_, and as a consequence the H_2_ flux necessary (qH_2_) but not the biomass yield on carbon.

### Effect of the residual formic acid concentration on the biomass yield

To study the effect of increasing residual formic acid concentration on the biomass production yield, a pH-controlled fed-batch cultivation was developed. The fermentation process was fully automated and required neither manual addition of formic acid nor nitrogen. Using data from preliminary cultures (not shown) with 2 g l^−1^ initial formic acid (pH 6.5), the composition and pH of the feeding solution was calculated and optimized. The aim was to maintain a constant concentration of formic acid and a sufficient supply of nitrogen based on the elementary composition of *R. eutropha*. The feeding solution contained 50% (w/v) formic acid solution employed with 4.3 g l^−1^ (252 mM) NH_3_(aq).

**Figure 2:**
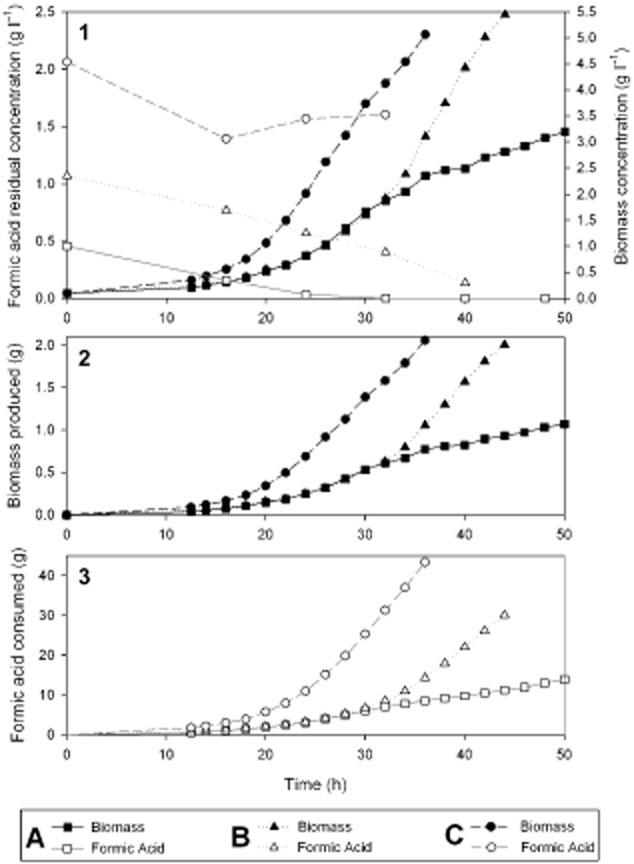
Growth of *R. eutropha* in a pH-controlled fed batch fermentation with formic acid as the sole substrate. Three different initial concentrations (A: 0.5 g l^−1^; B: 1.0 g l^−1^ and C: 2.0 g l^−1^) of formic acid were used to initiate the pH-controlled feeding. One culture for each initial concentration is depicted in the figure. The experiments were performed in duplicate.1. Biomass and residual formic acid concentrations over time.2. Total biomass produced over time.3. Total formic acid consumed over time.

Three different initial concentrations of pH-corrected formic acid (pH 6.5): 0.5 g l^−1^ (A), 1.0 g l^−1^ (B) and 2.0 g l^−1^ (C) were used to initiate the pH-controlled feeding (Fig. 2). Each culture was performed in duplicate.

When starting with 2.0 g l^−1^ pH-corrected formic acid (cultures C), concentrations of formic acid between 1.3 g l^−1^ and 2.1 g l^−1^ and a non-limiting nitrogen concentration were maintained during the growth phase (0–32 h). For the fermentations, which were initiated with 1.0 g l^−1^ of pH-corrected formic acid (cultures B), a decreasing residual concentration of formic acid over time was observed. When starting with an initial formic acid concentration of only 0.5 g l^−1^ (cultures A), the concentration in the medium decreased more rapidly with undetectable residual formic acid after 32 h leading to a linear growth phase.

A biomass amount of 1.98 ± 0.06 gCDW was reached for both cultures B and C, respectively, at 44 h and 36 h (Fig. 2.2). For the cultures B, the corresponding biomass concentration was 5.42 ± 0.04 gCDW l^−1^. For the cultures C, which were initiated with 2 g l^−1^ of formic acid, a lower biomass concentration of 4.89 ± 0.24 gCDW l^−1^ was achieved. To produce the same amount of biomass, a higher amount of formic acid was fed during the cultures C than during the cultures B (Fig. 2.3). This is due to a reduced biomass yield in the presence of higher formic acid concentration (see paragraph below). For the cultures A, the total amount of biomass produced was only 1.06 ± 0.02 g after 50 h, likely due to a carbon limitation as pointed out by the entrance in a linear growth phase at 32 h when the residual formic acid concentration was zero.

The biomass yield (Y_x_) was calculated for the growth phase of the fed-batch cultures (16–32 h).

**Figure 3:**
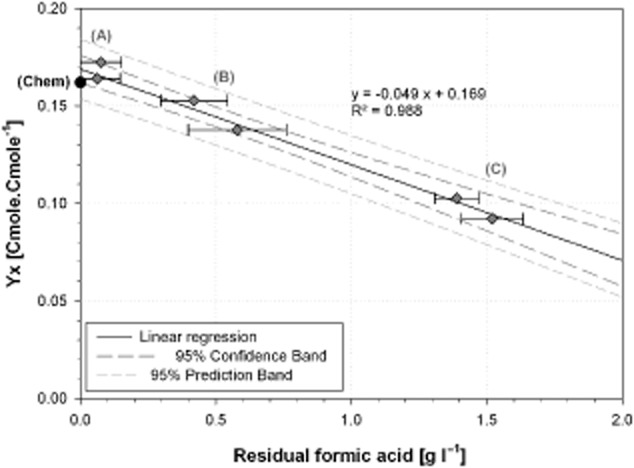
Effect of the formic acid concentration on the biomass yield (Y_x_). (♦): biomass yields of each fed-batch culture calculated during the growth phase between 16 h and 32 h for the pH-controlled fed-batch cultivation. (•): biomass yield calculated from data of the chemostat cultivation.

The highest biomass yields of 0.17 and 0.16 Cmole Cmole^−1^ (Fig. 3) were observed for the cultures A, with low formic acid concentrations between 0 g l^−1^ and 0.16 g l^−1^. These maximum growth yields are in accordance with the maximum experimental yield found during the chemostat culture (0.16 ± 0.01 Cmole Cmole^−1^) even though these three experiments were done in two different laboratories with different mineral media and culture systems.

Figure 3 presents the effect of the formic acid concentration on the biomass yield. A linear decrease in the biomass yield with increasing formic acid concentrations was observed. The experimental yield at null residual formic acid concentration was evaluated at 0.169 Cmole Cmole^−1^ with a 95% confidence interval of 0.162–0.176 Cmole Cmole^−1^. This confidence interval corresponds to 77–93% of the maximal theoretical yield, showing a high accordance between our experimental results and the theory and validated the maximum biomass yield from formic acid as substrate.

For the cultures B, the biomass yields were 0.14 and 0.15 Cmole Cmole^−1^ for average formic acid concentrations between 16–32 h of 0.58 ± 0.18 g l^−1^ and 0.42 ± 0.12 g l^−1^ respectively.

For the highest residual concentrations of formic acid of 1.39 ± 0.08 g l^−1^ and 1.52 ± 0.11 g l^−1^ (cultures C), the biomass yields were respectively 0.10 Cmole Cmole^−1^ and 0.09 Cmole Cmole^−1^.

A reduction of the biomass yield upon an increase in the formic acid concentrations from 2 g l^−1^ to 5 g l^−1^ was shown by Lee and colleagues (2006) for flask cultures. These cultures were first grown on 2 g l^−1^ glucose before the addition of a pulse of pH-corrected formic acid (pH 6.5–7.0) after 24 h (Lee *et al*., 2006). They showed a decrease in the biomass yield from approximately 0.08 gCDW gformic acid^−1^ (≈ 0.15 Cmole Cmole^−1^) to 0.05 gCDW gformic acid^−1^ (≈ 0.09 Cmole Cmole^−1^). However, their experimental yields were based on a single pulse of pH-corrected formic acid after heterotrophic growth on glucose and thus were not comparable to our yields. Our results were also in accordance with a previous study showing a clear decrease in the growth yields on other organic acids such as acetic acid and butyric acid (Grousseau, 2012).

If the linear correlation stands true also for higher formic acid concentrations, the biomass yield would approach zero at a concentration between 3.0 g l^−1^ and 4.1 g l^−1^. (95% confidence band; Fig. 3)

With a maximal biomass yield of 0.17 Cmole Cmole^−1^ obtained from the two cultivation conditions (chemostat and fed-batch), the highest biomass yield for growth of *R. eutropha* published so far was reached. Friedrich and colleagues (1979) evaluated the biomass production yield to 2.35 g.mole^−1^ which corresponded to 0.09 Cmole Cmole^−1^; no indication concerning the residual formic acid concentration was given.

### Determination of the maximum growth rate on formic acid

The fed-batch culture was also a pertinent tool to study the growth dynamic and was used to determine the maximum growth rate. The highest maximal growth rate μ_max_ of 0.18 h^−1^ ± 0.00 was reached during the fed-batch cultures C (exponential growth phase of the cultures from 16 h to 24 h). Friedrich and colleagues (1979) determined a growth rate of 0.17 h^−1^ and Friedebold and Bowien (1993) a growth rate of 0.23 h^−1^ for the PHB producing *R. eutropha* wild-type strain (H16). However, since PHB contributes to the CDW as well as to the OD_600_ value, the determined growth rate in these two articles could have been overestimated.

Nonetheless, those growth rates are low compared to growth rates measured for other organic acids like acetic, propionic or butyric acid, for which growth rates between 0.26 h^−1^ and 0.34 h^−1^ can be reached (Kim *et al*., 1992; Wang and Yu, 2000; Grousseau, 2012) or for lithoautotrophic growth with growth rates determined between 0.31 h^−1^ and 0.32 h^−1^ (Schlegel *et al*., 1961; Siegel and Ollis, 1984). The low growth rate of 0.18 h^−1^ could be explained by a limiting energetic flow since lithoautotrophic growth exhibited a higher growth rate. As explained in section *Determination of the maximal biomass production yield in formic acid limited continuous culture*, the NADH supply seemed to be the limiting factor for the carbon assimilation by the CBB cycle. With formic acid, carbon and energy sources are coupled, in contrast to lithoautotrophic growth where H_2_ supplies the energy and CO_2_ the carbon.

### Conclusion

In this study, two different tools to explore the organoautotrophic growth of *R. eutropha* on formic acid were used. In both systems, biomass concentration was high enough to provide reliable quantitative information (10.6 g l^−1^ and 5.4 g l^−1^).

A chemostat culture was used to determine the maximum biomass yield in a stabilized system at a null residual formic acid concentration to avoid toxic effects. This maximum biomass yield was confirmed by the fed-batch study. A good adequation between experimental and theoretical values was shown. The experimental yield of 0.17 Cmole Cmole^−1^ corresponded to 81% to 89% of the maximal theoretical yield.

pH-controlled fed-batch cultures were used to investigate the effect of increasing concentration of formic acid: a decrease in biomass production yield with increasing residual formic acid concentration was shown between 0.0 g l^−1^ and 1.5 g l^−1^. This dynamic system was also used to determine the maximum growth rate of the strain on formic acid which was equal to 0.18 h^−1^.

### Experimental procedures

The physiological characterization of *R. eutropha* strain Re2061 was carried out in two distinct research laboratories: the Fermentation Advances and Microbial Engineering group of Laboratoire d'Ingénierie des Systèmes Biologiques et des Procédés at the Institut National des Sciences Appliquées of Toulouse (France) for the chemostat culture and the Sinskey Lab, Biology department of Massachusetts Institute of Technology (USA) for the pH-controlled fed-batch cultures. Materials and equipments were therefore lab dependent.

#### Bacterial strain

The recombinant *R. eutropha* strain Re2061 (H16Δ*phaCAB* Gen^r^) unable to produce PHB was used in the two laboratories. This strain was engineered from strain H16 (ATCC 17699) by eliminating the *phaCAB* operon, which encodes the polymer biosynthesis enzymes β-ketothiolase, acetoacetyl-CoA reductase and PHB synthase, from the *R. eutropha* genome and was first described by Lu and colleagues (2012). It was selected as a platform strain for further metabolic engineering.

#### Continuous culture experiment

##### Growth media

Rich medium consisted of 2.5 g l^−1^ tryptone, 2.5 g l^−1^ meat peptone and 3 g l^−1^ meat extract. The media used for the pre-cultures was described in the literature by Aragao and colleagues (1996) and adapted by Grousseau and colleagues (2013) except for the carbon source (fructose added to a final concentration of 4 g l^−1^). For the continuous fermentation, the culture medium was the mineral salt medium adapted by Gaudin (1998). The carbon source was formic acid. These growth media were designed and optimized to supply all nutritional elements required to produce a biomass concentration of about 10 g l^−1^.

##### Pre-culture cultivation

A single colony grown on a rich medium (with addition of agar 15 g l^−1^) petri dish was used to inoculate 10 ml of rich medium, which was incubated for 24–48 h at 30°C. The second and the third pre-cultures were respectively grown for 24 h and 18 h, at 30°C, in a baffled 1 l shaking flask (150 rpm) and a baffled 3 l shaking flask (120 rpm), containing 150 ml and 300 ml of pre-culture mineral salt medium with fructose as carbon source.

The cell suspension of the third pre-culture was used to inoculate the fermenter to an initial biomass concentration of 0.1 g l^−1^.

##### Continuous cultivation

The continuous culture was performed in a 7 l fermenter (BIOSTAT B-DCU, Sartorius Stedim Biotech, Germany) with a working volume of 2.7 l, equipped with pH, dissolved oxygen (DO), temperature, pressure and anti-foam controllers. The online monitoring and control systems of the reactor were handled by the software biopat mfcs/win version 3.0. The DO level in the reactor was controlled above 20% of air saturation by varying stirring speed and/or inlet air flow rate. Temperature was maintained at 30°C. The pH was maintained at 7.0 by addition of a NH_4_OH solution with a concentration of 8.24 mol l^−1^.

A batch culture with fructose as carbon source was carried out to initiate the growth and to quickly reach a biomass concentration close to 10 g l^−1^. After fructose exhaustion (35 h), the continuous culture was started with a dilution rate of 0.05 h^−1^ using a 98% (w/v) formic acid solution as sole substrate to maintain a biomass concentration around 10 g l^−1^. Added masses of substrate, mineral salt medium and NH_4_OH were online monitored by weight. Inlet and outlet gases were analysed using a gas analyser with an infrared spectrometry detector for carbon dioxide and a paramagnetic detector for oxygen (EGAS-8 gas analyser system; B. Braun Biotech International, Germany). Dioxygen consumption rate and carbon dioxide production rate were calculated from mass balances, taking into account the evolution of inlet airflow, temperature and pressure.

#### pH-controlled fed-batch experiments

##### Growth media

Rich medium consisted of 27.5 g l^−1^ dextrose-free tryptic soy broth (TSB) (Becton Dickinson, Sparks, MD). Minimal medium was formulated as described previously (Budde *et al*., 2010). All cultures contained 10 μg ml^−1^ of gentamicin sulfate. Amounts of added carbon and nitrogen are described in *Pre-culture cultivation and pH-controlled fed-batch cultivation*.

##### Pre-culture cultivation

A single colony grown on a TSB agar plate was used to inoculate 5 ml of TSB medium, which was incubated at 30°C for 24 h on a roller drum. A baffled 250 ml shaking flask, containing 50 ml minimal medium with 17 g l^−1^ of fructose and 2.3 g l^−1^ of urea, was inoculated with 1% (v/v) of the overnight culture. The flask culture was incubated at 30°C, shaking at 200 rpm for 24 h. Afterwards, cells were centrifuged at 5000 × *g* for 10 min, and the cell pellet was suspended in 0.85% saline to remove residual amounts of carbon and nitrogen. The cell suspension was used to inoculate the fermenters to an initial biomass concentration of 0.1 g l^−1^.

##### pH-controlled fed-batch cultivation

A Multifors Bioreactor System (Infors AG, Switzerland) consisting of six vessels with a 500 ml working volume was used for the fed-batch cultures.

The initial volume was 350 ml of minimal medium with 1 g l^−1^ of NH_4_Cl. Approximately 0.060 g l^−1^ (∼ 3 drops) of polypropylene glycol P2000 were added to avoid foam formation.

Control set point for temperature was 30°C. The pH was set to 6.7 ± 0.1. Gas flow was set to 2 vvm. The dissolved oxygen concentration was maintained at 25% using a two-level cascade. At the first level, the stirrer speed was set to increase up to 1000 rpm. After that, a second level was employed to add pure oxygen in increasing time intervals by switching between airflow (2 vvm) and O_2_ flow (0.3 vvm).

A pH-controlled feeding was used to add formic acid and nitrogen source in small increments. A 50% (w/v) formic acid solution containing 4.3 g l^−1^ (252 mM) of NH_3_(aq) was used as the feed. The principle of a pH-stat consists in the addition of the formic acid as the carbon and energy substrate via the bioreactor pH controller in response to a pH decrease linked to the consumption of formic acid by the cells. The initial concentrations of formic acid were adjusted by using a solution of 20% (w/v) formic acid solution, pH corrected with NaOH to pH 6.5. The amount of added feed medium was determined by weight. Three different initial concentrations of formic acid were tested: cultures A 0.5 g l^−1^, cultures B 1.0 g l^−1^ and cultures C 2.0 g l^−1^. The initial pH of the fermentation medium was corrected to the set point of pH 6.7 by titration with 1 M NaOH. The experiments were performed in duplicate. Six fed-batch cultures have been carried out.

#### Analytics

##### Determination of ammonia concentrations

Ammonia concentration was determined enzymatically using a commercial Ammonia Assay Kit (Sigma-Aldrich). Culture samples were centrifuged in a tabletop centrifuge at 16 000 × *g* for 2 min, and the supernatant was diluted and analysed according to the manufacturer's instructions.

##### Determination of metabolite concentrations

Culture supernatant was obtained by centrifuging (Mini-Spin Eppendorf, USA) the fermentation broth in Eppendorf tubes at 13 000 rpm for 3 min. The supernatant was filtered on Minisart filters 0.20 μm pore-size diameter polyamide membranes (Sartorius AG, Germany).

The culture supernatants were analysed by high performance liquid chromatography (DIONEX Ultimate 3000, USA or Agilent 1100 Series) using an Aminex HPX-87H^+^column (Bio-RAd, USA) and its guard column (Micro-Guard Cation H, Bio-Rad, 4.6 × 30 mm) and the following conditions: a temperature of 50°C with 5 mM H_2_SO_4_ as eluent at a flow rate of 0.6 ml min^−1^ or 2.5 mM H_2_SO_4_ at 0.5 ml min^−1^ and a dual detection (RI and UV at 210 nm).

For quantification of low formic acid concentrations, the samples were analysed by high performance ion chromatography [ICS-3000 system (Dionex) equipped with an ED40 electrochemical detector]. Formic acid was separated on an IonPac AS11-HC analytical (4 mm × 250 mm) as described in Sunya and colleagues (2012) except for the gradient which was self-generated as follow: 0–13 min, KOH 1 mM; 13–25 min, the gradient increased linearly from 1 mM to 15 mM KOH, raised stepwise to 30 mM from 25 min to 35 min, then to 60 mM from 35 min to 45 min and kept at this concentration from 45 min to 50 min and finally decreased to 1 mM for 5 min.

##### Determination of biomass concentrations

Biomass concentration was determined photometrically at an OD_600_ using a spectrophotometer (BIOCHROM LIBRA S4 or Spectronic GENESYS 20 Visible Spectrophotometer). Cell dry weight was determined gravimetrically after separation of the cells from the broth, washing of the cell pellet and complete dehydration in an oven under vacuum.

#### Calculations and metabolic descriptor

All yields were expressed as carbon ratios in Cmole Cmole^−1^. For the fed-batch cultures, the experimental biomass yield (*Y_X_*) was calculated using the equation *Y_X_* = (*X*_2_ − *X*_1_)/(*MW_X_*(*S*_2_ − *S*_1_)) where X was the mass of cells (g cell dry weight) produced and S the mass of substrate (Cmole) consumed within a time interval t_2_–t_1_ and *MW_X_* the molecular weight of *R. eutropha* per Cmole, which is 25.35 g Cmole^−1^ (Aragao, 1996). The masses were calculated taking into account the evolution of the suspension volume due to the feed and the sampling volumes.

For both cultures, the specific substrate (formic acid) uptake rate (*q_S_*) and growth rate (μ) were calculated from their measured data by means of the respective mass balance equation, taking into account the evolution of the suspension volume.

Moreover, for the continuous culture, CO_2_ production and O_2_ consumption were measured with the gas analyser (*Continuous cultivation*) allowing to perform a data reconciliation based on carbon and reduction degree balances (van der Heijden *et al*., 1994a,b).

A metabolic descriptor (Grousseau *et al*., 2013) was used to calculate theoretical values of biomass yield (Y_X_^theo^), RQ, specific oxygen uptake rate and specific dioxide carbon production rate. The model was implemented with three reactions concerning formic acid catabolism: